# E-cigarette Unit Sales by Product and Flavor Type, and Top-Selling Brands, United States, 2020–2022

**DOI:** 10.15585/mmwr.mm7225a1

**Published:** 2023-06-23

**Authors:** Fatma Romeh M. Ali, Andrew B. Seidenberg, Elisha Crane, Elizabeth Seaman, Michael A. Tynan, Kristy Marynak

**Affiliations:** ^1^CDC Foundation, Atlanta, Georgia; ^2^Truth Initiative, Washington, DC; ^3^Office on Smoking and Health, National Center for Chronic Disease Prevention and Health Promotion, CDC.

E-cigarette products, related policies, and use patterns change rapidly. In the United States, the prevalence of e-cigarette use is markedly higher among youths and young adults than it is among adults overall. In 2021, 4.5% of all adults aged ≥18 years (an estimated 11.1 million) and 11.0% of young adults aged 18–24 years (an estimated 3.1 million) currently (≥1 day during the previous 30 days) used e-cigarettes; during 2022, 14.1% of high school students (an estimated 2.14 million) currently used e-cigarettes (*1*,*2*). E-cigarettes often contain high concentrations of nicotine. Nicotine is highly addictive and can harm the adolescent brain, which continues to develop through approximately age 25 years (*3*). Since 2020, the availability of e-cigarette products has changed in response to multiple factors, including local and state policies to address flavored e-cigarette sales, actions undertaken by the Food and Drug Administration (FDA), COVID-19–related closures, and global supply chain disruptions. To assess trends in unit sales of e-cigarettes in the United States, by product and flavor, and top-selling brands, the CDC Foundation, Truth Initiative,* and CDC analyzed retail scanner data during January 26, 2020–December 25, 2022, from Information Resources, Inc. (IRI), a U.S. data analytics and market research company. Overall, unit sales increased by 46.6% during the study period. The unit share of menthol-flavored product sales remained relatively stable during this period, whereas nonmenthol flavor unit shares changed. During January 26, 2020–December 25, 2022, unit shares of tobacco-flavored and mint-flavored products decreased (from 28.4% to 20.1% and from 10.1% to 5.9%, respectively), whereas shares of other flavor sales increased (from 29.2% to 41.3%). In addition, during January 2020–December 2022, unit shares of prefilled cartridges decreased from 75.2% to 48.0%, and disposable e-cigarette unit share increased from 24.7% to 51.8% of total unit sales. The five top-selling e-cigarette brands for the 4-week period ending December 25, 2022, were Vuse, JUUL, Elf Bar, NJOY, and Breeze Smoke. Analysis of information on e-cigarette retail sales can guide strategies to prevent youth access to and use of e-cigarettes, including restrictions on flavored tobacco products (*4*).

U.S. e-cigarette sales data were licensed from IRI, which included Universal Product Code sales from brick-and-mortar retailers only; sales from online retailers and tobacco specialty stores, including vape shops, were not available. For analyses other than top-selling brands, e-cigarette products were categorized as prefilled cartridges, disposable devices, or e-liquids^†^ (*5*), and e-cigarette accessories and devices sold without e-liquids (accounting for 9.5% of sales) were excluded. Product flavor names were categorized as tobacco, menthol, mint, or all other flavors (e.g., fruit, clove or spice, candy, desserts, other sweets, chocolate, alcoholic and nonalcoholic drinks). Ambiguous or concept flavors (e.g., “fusion”), which constituted 5.6% of sales, were searched online and back-coded into one of the four flavor categories. E-cigarette unit sales were standardized and summed during 4-week periods during January 26, 2020–December 25, 2022^§^. Analyses were performed for total unit sales and the proportion of total unit sales (unit share) by flavor and product type using Stata (version 17; StataCorp). Trends during the analysis period were analyzed using Joinpoint regression (version 4.9.1.0; National Cancer Institute), which detects points in time when changes in trend (or slope changes) are statistically significant. The average 4-week period percentage change (APPC) was calculated as the average of the slope coefficients of the Joinpoint regression line. P-values <0.05 were considered statistically significant. Total number of brands and a list of the top five brands with the highest unit sales, as provided in the IRI database without unit standardization or exclusions, were reported for the beginning and end of the study period. This study was reviewed by CDC and was conducted consistent with federal law and CDC policy.^¶^

During January 2020–December 2022, total U.S. e-cigarette unit sales increased by 46.6%, from 15.5 million to 22.7 million units per 4-week period (APPC = 1.1; p<0.05); however, sales fluctuated during this period (Figure 1). Although sales increased during January 2020–May 2022, the percentage of increase in sales slowed from 36.5% (15.5 million to 21.2 million; APPC = 6.9) during January 2020–June 2020 to 16.8% (21.2 million to 24.7 million; APPC = 1.3) during June 2020–June 2021 to 4.9% (24.7 million to 25.9 million; APPC = 0.4) during June 2021–May 2022 (p<0.05 for all APPCs). Overall, during January 2020–May 2022, total sales increased 67.2% (APPC = 1.8; p<0.05), from 15.5 million to 25.9 million units per period. During May–December 2022, total sales decreased by 12.3% (APPC = –1.8; p<0.05), to 22.7 million units per period.

**FIGURE 1:**
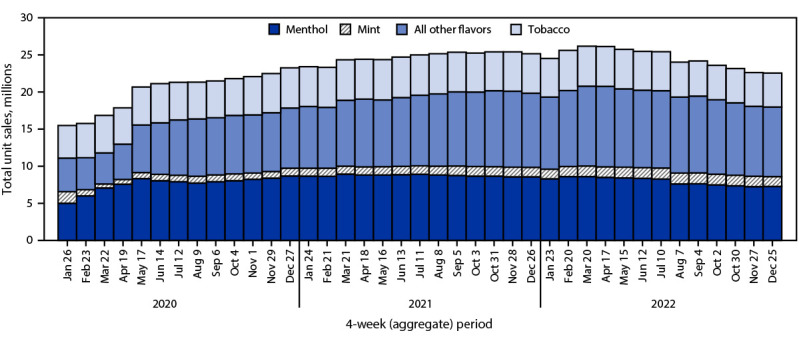
Total e-cigarette unit sales,* by flavor^†^ — United States, January 26, 2020–December 25, 2022 * Retail sales data obtained from Information Resources, Inc. for convenience stores, gas stations, grocery stores, drug stores or pharmacies, mass merchandiser outlets, club stores, dollar stores, and military sales; Internet and vape shop sales were not recorded. ^†^ The “All other flavors” category includes fruit, clove or spice, chocolate, alcoholic drink (such as wine, cognac, or other cocktails), candy, desserts, other sweets, or some other flavor. Unknown flavors were excluded from this figure (<0.1%).

Among total e-cigarette unit sales during January 2020–December 2022, the percentage of menthol flavor sales did not significantly change (<1%, from 32.3% in January 2020 to 31.9% in December 2022), whereas the percentages of tobacco, mint, and other flavor sales fluctuated. During the period of increasing total sales (January 2020–May 2022), decreases were observed in the percentages of sales of both tobacco flavor (from 28.4% to 20.5%; APPC = –1.1; p<0.05) and mint flavor e-cigarettes (10.1% to 5.6%; APPC = –1.9; p<0.05), while the percentage of other flavor sales increased from 29.2% to 40.8% (APPC = 1.1; p<0.05). During the period of declining total sales (May–December 2022), the percentage of sales of tobacco-flavored e-cigarettes decreased slightly, from 20.5% to 20.1% (APPC = –0.6; p<0.05), while slight increases in sales of mint-flavored (from 5.6% to 5.9%) and other-flavored e-cigarettes (40.8% to 41.3%) occurred (APPC = 1.3 and 0.4, respectively; p<0.05).

Among total e-cigarette unit sales during January 2020–December 2022, the percentage of prefilled cartridge sales decreased from 75.2% to 48.0% (APPC = –1.1; p<0.05). In contrast, the percentage of disposable e-cigarette sales more than doubled, from 24.7% in January 2020 to 51.8% in December 2022 (APPC = 1.9; p<0.05). Among prefilled cartridge e-cigarettes sales in January 2020, tobacco, menthol, and mint flavors accounted for 34.2%, 40.0%, and 10.5% of sales, respectively, whereas e-cigarette sales of other flavors accounted for 15.3% (Figure 2). During December 2022, the prefilled cartridge market was composed of tobacco- (37.3%) and menthol- (62.2%) flavored sales almost exclusively. Among disposable e-cigarette sales during January 2020, tobacco, menthol, mint, and other flavors accounted for 10.5%, 9.0%, 8.9%, and 71.4%, respectively (Figure 3). By December 2022, the disposable e-cigarette market was led by mint (11.1%) and flavors other than tobacco, menthol, or mint (79.6%); tobacco- and menthol-flavored sales accounted for 4.3% and 3.6%, respectively.

**FIGURE 2:**
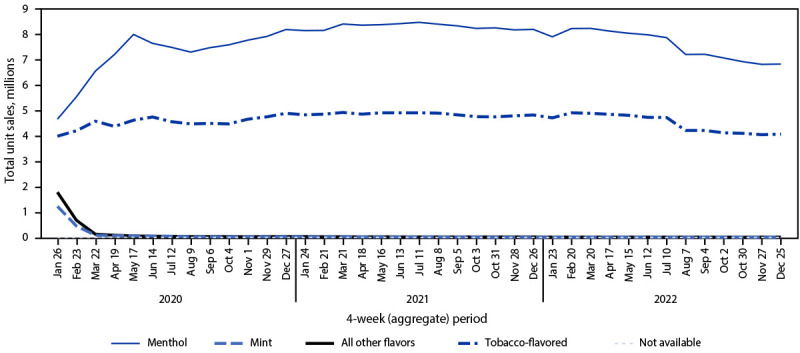
Prefilled cartridge* e-cigarette unit sales,^†^ by flavor^§^ — United States, January 26, 2020–December 25, 2022 * Prefilled cartridges include tanks, cartridges, and pods used in rechargeable and reusable e-cigarette devices; the cartridges are not intended to be refilled after the liquid has been depleted. Unit sales were standardized to reflect the most common package size for each product type; a standardized unit was equal to five prefilled cartridges. ^†^ Retail sales data obtained from Information Resources, Inc. for convenience stores, gas stations, grocery stores, drug stores or pharmacies, mass merchandiser outlets, club stores, dollar stores, and military sales; Internet and vape shop sales were not recorded. ^§^ The “All other flavors” category includes fruit, clove or spice, chocolate, alcoholic drink (such as wine, cognac, or other cocktails), candy, desserts, other sweets, or some other flavor.

**FIGURE 3:**
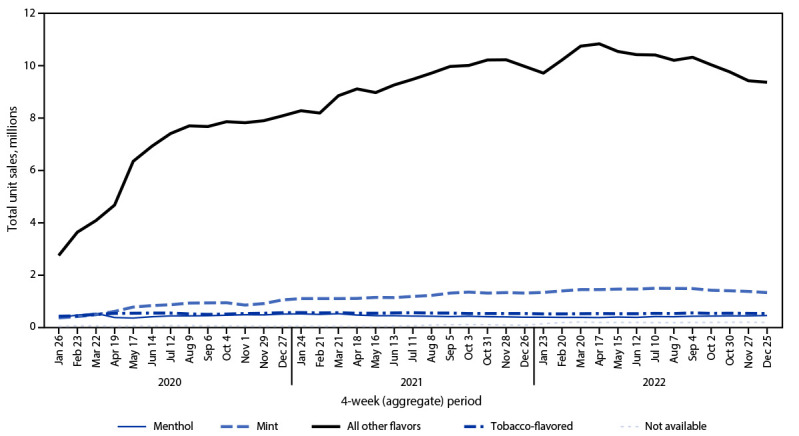
Disposable e-cigarette* unit sales,^†^ by flavor^§^ — United States, January 26, 2020–December 25, 2022 * Disposable devices include nonrechargeable and nonreusable e-cigarette devices that are not intended to be refilled with e-liquid after being depleted; the device is disposed of once the e-liquid has been consumed. Unit sales were standardized to reflect the most common package size for each product type; a standardized unit was equal to one disposable device. ^†^ Retail sales data obtained from Information Resources, Inc. for convenience stores, gas stations, grocery stores, drug stores or pharmacies, mass merchandiser outlets, club stores, dollar stores, and military sales; Internet and vape shop sales not captured. ^§^ The “All other flavors” category includes fruit, clove or spice, chocolate, alcoholic drink (such as wine, cognac, or other cocktails), candy, desserts, other sweets, or some other flavor.

During the 4-week period ending January 26, 2020, among 184 brands, the top five in descending order of sales were JUUL, Vuse, NJOY, My Blu, and Puff.** During the 4-week period ending December 25, 2022, the top five brands were Vuse, JUUL, Elf Bar, NJOY, and Breeze Smoke. The total number of e-cigarette brands increased 46.2% during the study period, from 184 to 269. Vuse, JUUL, NJOY, and My Blu are prefilled cartridge brands; Puff, Elf Bar, and Breeze Smoke are disposable.

## Discussion

E-cigarette unit sales during December 2022 were 46.6% (7.2 million units) higher than sales during January 2020. Declines in total unit sales observed during May 2022–December 2022 likely reflect multiple factors, including local and state restrictions on flavored tobacco product sales, FDA regulatory actions, potential COVID-19–associated supply chain disruptions, inflation, and a recent proliferation of large format disposable e-cigarettes capable of delivering thousands of “puffs” that might permit higher nicotine consumption per unit. Increases in the number of available e-cigarette brands during the study period and changes observed in the top five brands during December 2022 reflect the dynamic nature of the e-cigarette market.

Citing the appeal of flavored e-cigarettes to children, FDA announced during January 2020 that it would prioritize enforcement against prefilled e-cigarettes in flavors other than tobacco and menthol based on the prevalence of use of these products among youth at the time.^ ††^ The present study’s findings indicate that after this announcement, retail sales of mint- and other- flavored prefilled cartridges halted while notable increases in sales of fruit- and mint-flavored disposable products occurred. Although disposable e-cigarettes constituted approximately less than one quarter of total unit sales during January 2020, disposable sales surpassed refillable sales in March 2022. As of August 2022, Elf Bar, a disposable brand that has driven sharp increases in e-cigarette use among persons aged 16–19 years in England, is the top disposable brand reported among a sample of 4,142 persons aged 16–19 years in the United States (*6*) and was the top-selling disposable brand in December 2022. In addition, flavored disposable e-cigarettes have emerged as the most commonly used device type among U.S. middle and high school students who use e-cigarettes (*1*). These sales data, coupled with behavioral data, demonstrate that the e-cigarette landscape and use patterns rapidly shift in response to market changes, policy interventions, and other factors.

As of December 31, 2022, seven states (California, Maryland, Massachusetts, New Jersey, New York, Rhode Island, and Utah) and 378 jurisdictions, including counties, cities, towns, and villages, have some type of restriction on flavored e-cigarette sales in place. The comprehensiveness of local and state flavored tobacco product policies varies (*7*), with some policies exempting certain flavors (e.g., menthol) or products (e.g., cigars), which are disproportionately used by certain groups such as non-Hispanic Black or African American youths (*1*). States such as Massachusetts, which have well-enforced comprehensive flavor restrictions, have experienced large and sustained declines in total e-cigarette sales (*8*). Further, a review of nine studies found that after a flavored tobacco product sales restriction, use of tobacco products among young persons declined *(9)*. The trends observed nationally in the relative proportions of disposable e-cigarette sales are observable within states lacking e-cigarette flavor restrictions.^ §§ ^

Through the premarket tobacco application process established by the Tobacco Control Act, FDA can authorize or deny the marketing of tobacco products using the standard that allowing the product to be marketed is “appropriate for the protection of public health.”^ ¶¶^FDA issued its first marketing denial orders for approximately 55,000 flavored e-cigarette products on August 26, 2021, and its first marketing denial order for a menthol-flavored, cartridge-based e-cigarette on October 26, 2022***; to date, only tobacco-flavored e-cigarette products have received marketing authorization on the basis of a scientific evaluation of their risks and benefits to the population as a whole.^†††^ FDA has taken action to address illegal flavored disposable e-cigarette products, including the issuance of warning letters to importers, distributors, and retailers for the unauthorized sale of Puff Bar products, the most commonly used e-cigarette brand among U.S. middle and high school students in 2022 (*10*). Additional FDA enforcement efforts against manufacturers or retailers include no-tobacco-sale orders, permanent injunctions against noncompliant manufacturers in conjunction with the U.S. Department of Justice, and other actions.^§§§^

The findings in this report are subject to at least three limitations. First, sales data from tobacco specialty stores, including vape shops and internet retailers, were not available. However, online sales are estimated to constitute only 20% of total e-cigarette sales (*4*). Second, these analyses did not account for variations in e-cigarette nicotine strength or unit size. Large-format disposable e-cigarettes, including (but not limited to) Elf Bar BC5000, have recently been introduced. Therefore, recent declines in unit sales might not signify declines in consumption. Finally, purchaser age is not available from IRI. Sales reflect purchases by adults and could also reflect direct or indirect purchases by youths.

Comprehensive restrictions on the sale of all flavored tobacco products that include e-cigarettes, menthol cigarettes, and flavored cigars are warranted in all jurisdictions. These strategies, when coupled with longstanding evidence-based strategies to prevent youth tobacco use such as price increases, comprehensive smokefree policies that include e-cigarettes, and counter-marketing campaigns, are expected to reduce youth initiation and use as well as reduce disparities in tobacco product use.

**Summary**

**What is already known about this topic?**

E-cigarette products, related policies, and use patterns change rapidly. Flavored e-cigarette products appeal to young users.

**What is added by this report?**

E-cigarette unit sales increased by 46.6% during January 2020–December 2022. After January 2020, sales of mint and other flavored prefilled cartridges ceased, and disposable e-cigarettes in fruit, sweet, and other flavors increased. Disposable e-cigarettes in youth-appealing flavors are now more commonly sold than prefilled units.

**What are the implications for public health practice?**

Monitoring e-cigarette sales can inform strategies to prevent youth tobacco use, including restrictions on flavored tobacco products.

## Footnotes

* https://truthinitiative.org/

† Prefilled cartridges include tanks, cartridges, and pods used in rechargeable and reusable e-cigarette devices; the cartridges are not intended to be refilled after the liquid has been depleted. Disposable devices include nonrechargeable and nonreusable e-cigarette devices that are not intended to be refilled with e-liquid after being depleted; the device is disposed of once the e-liquid has been consumed. E-liquids are containers of the liquid used in e-cigarette devices, which typically contains a humectant (e.g., propylene glycol), nicotine, and flavoring.

§ Consistent with previous studies, unit sales were standardized to reflect the most common package size for each product type. A standardized unit was equal to five prefilled cartridges, one disposable device, or one e-liquid bottle.

¶ 45 C.F.R. part 46, 21 C.F.R. part 56; 42 U.S.C. Sect. 241(d); 5 U.S.C. Sect. 552a; 44 U.S.C. Sect. 3501 et seq.

** Individual brands (reported by IRI as “brand franchises”) might include multiple product lines (e.g., Vuse includes the product lines Vuse Alto and Vuse Solo, Elf Bar includes Elf Bar BC5000 and Elf Bar, and Puff includes Puff Bar).

††
https://www.fda.gov/news-events/press-announcements/fda-finalizes-enforcement-policy-unauthorized-flavored-cartridge-based-e-cigarettes-appeal-children

§§ https://www.federalregister.gov/documents/2021/10/05/2021-21011/premarket-tobacco-product-applications-and-recordkeeping-requirements

¶¶
https://www.fda.gov/news-events/press-announcements/fda-denies-marketing-applications-about-55000-flavored-e-cigarette-products-failing-provide-evidence

*** https://www.cdcfoundation.org/State-E-CigaretteSales-DataBrief-2022-Octo30?inline

†††
https://www.fda.gov/tobacco-products/premarket-tobacco-product-applications/premarket-tobacco-product-marketing-granted-orders

§§§
https://www.fda.gov/tobacco-products/compliance-enforcement-training/ctp-compliance-enforcement
